# Biomarkers with Therapeutic or Prognostic Applications in Gynecologic Malignancies

**DOI:** 10.3390/cancers18081248

**Published:** 2026-04-15

**Authors:** Mohamed Mokhtar Desouki, Katherine L. Mager

**Affiliations:** 1 Department of Pathology, Roswell Park Comprehensive Cancer Center, Buffalo, NY 14203, USA; 2 Jacobs School of Medicine and Biomedical Sciences, State University of New York at Buffalo, Buffalo, NY 14203, USA; 3 Department of Gynecologic Oncology, Roswell Park Comprehensive Cancer Center, Buffalo, NY 14203, USA; katherine.lavignemager@roswellpark.org

**Keywords:** ER, PR, Her2, MMR, P53, PD-L1, FOLR1, *POLE*, IHC, clinical trial

## Abstract

Background/Objectives: The treatment of gynecologic malignancies has moved towards a precision medicine model with an approach to prognostication and management based on biomarker testing. The objective of this review is to describe the current landscape of biomarker testing in gynecologic cancer including clinical implications and the approach to testing. Methods: A review of the literature was performed that included published clinical trials which utilized biomarker testing as part of inclusion/exclusion criteria, prospective trials that addressed the application and scoring of biomarkers utilized in gynecologic cancers, prospective clinical trials that utilized biomarker findings to determine management, and national or society guidelines for the scoring of biomarkers and treatment of gynecologic cancers. Findings: The use of biomarker testing as part of the management of gynecologic cancers is the standard of care for both treatment and prognostication. In endometrial cancer, biomarker testing has been incorporated into the staging system and impacts treatment in both the upfront and recurrent setting. Specific biomarkers of interest for endometrial cancer include estrogen receptor (ER), progesterone receptor (PR), DNA Polymerase Epsilon (*POLE*), mismatch repair proteins (MMR), and Human Epidermal growth factor Receptor-2 (HER2). In ovarian cancer, biomarker testing is primarily utilized in the recurrent setting to guide management of platinum-resistant ovarian cancer with a specific focus on targeted therapy utilizing antibody drug conjugates (ADCs) and immunotherapy. Immunotherapy has become an important component of therapy for cervical cancer and Programmed death-ligand 1 (PD-L1) testing is a key biomarker in determining treatment. Conclusions: The utilization of appropriate assays and processes to accurately assess the status of biomarkers in the pathology laboratory is crucial to the treatment of gynecologic malignancies in the precision medicine era.

## Simple Summary

Treatment of gynecologic cancer has moved to a precision medicine model with biomarkers now forming the foundation of both treatment and prognostication. These markers are assessed using multiple modalities, most commonly immunohistochemistry (IHC) and next-generation sequencing (NGS). IHC markers are widely utilized to determine the status of estrogen receptors (ER), progesterone receptors (PR), mismatch repair proteins (MMR), Human Epidermal growth factor Receptor-2 (HER2), Folate receptor alpha (FOLR1), and p53, among others. NGS is used routinely to determine DNA Polymerase Epsilon (*POLE*) status in the setting of endometrial cancer and is often utilized in the setting of clinical trial eligibility across gynecologic cancers. The utilization in the laboratory of appropriate assays and processes to accurately assess the status of these markers is crucial to the treatment of gynecologic malignancies.

## 1. Introduction

Testing tumors for the expression/loss of markers by different testing modalities such as immunohistochemistry (IHC) and molecular testing is a common practice in modern pathology and biomarker results have become the foundation of precision medicine in the treatment of gynecologic malignancies. These results provide crucial diagnostic, prognostic, and therapeutic information that facilitate the formulation of personalized treatment plans. There are many applications of biomarkers ranging from treatment to prognostication. An example of a biomarker guiding treatment is Human Epidermal growth factor Receptor-2 (HER2) testing to determine likelihood of response to HER2-targeted drugs such as trastuzumab. Other markers can be used to predict the response to immunotherapy or hormonal therapy such as Programmed death-ligand 1 (PD-L1) and steroid hormone receptors, such as estrogen (ER) and progesterone (PR) receptors, in certain tumors.

Markers are also used to predict the behavior and prognosis of tumors. An example of this is Ki-67, where high proliferation rates correlate with more aggressive behavior in most tumors. P53 IHC stain can determine if “mutant” expression is present, which is associated with a poor prognosis across tumor types [1]. Biomarkers are sometimes used to aid in diagnosis. Examples of this are HMB45, which is used for the diagnosis of uterine perivascular epithelioid cell tumors (PEComas) [2] or cytokeratins to determine the tissue of origin and the lineage of tumors. Extent of disease and monitoring of treatment effectiveness are other applications for biomarkers. IHC can detect small clusters of tumor cells which may not be apparent by routine H&E stains and, consequently, micrometastasis, or isolated tumor cells, could be identified which may influence treatment and have prognostic implications.

The treatment of gynecologic malignancies has moved towards a precision medicine model with a personalized approach to prognostication and management based on biomarkers. Biomarkers for endometrial, ovarian, and cervical cancers are now included in the National Comprehensive Cancer Network (NCCN) guidelines in the United States as well as other professional and international guidelines, allowing for broader use and for more accessible testing-based guidelines. Selecting a biomarker for clinical use depends on multiple parameters including the specificity, sensitivity, accessibility, and cost. This review will give an overview of the currently utilized biomarkers in gynecologic malignancies and will include the selection of biomarkers, approach to testing, and clinical application.

## 2. Methods

A review of the literature was performed in PubMed including English language publications between 2015 to 2026 with the keywords gynecologic tumors, markers, immunohistochemistry (IHC), and clinical trial. The publications selected for inclusion included trials that utilized one or more of the markers of interest [3]. The key trials utilizing biomarker testing in the treatment of gynecologic cancers have been summarized in Table 1.

## 3. Discussion/Observations

### 3.1. Estrogen (ER) and Progesterone (PR) Receptors

Steroid receptors, namely ER and PR, are often noted to be upregulated in gynecologic cancer and serve as a target in the treatment of endometrial cancer, uterine sarcoma, and a subset of ovarian cancer. They play an essential role in the pathogenesis of type I endometrial carcinomas, which is mostly seen arising in the background of endometrial hyperplasia.

#### 3.1.1. Clinical Applications

Uterine sparing treatment with progesterone can be considered for a subset of highly selected patients who desire fertility or in patients who are not candidates for aggressive management, such as surgery or cytotoxic chemotherapy. The agents typically used are high-dose progestins, either megesterol acetate or a levonorgestrel-releasing intrauterine device (IUD). Currently, a commonly used regimen is a levonorgestrel-releasing IUD due to its favorable side effect profile and high response rate [4,11].

In a prospective phase 2 trial, 57 patients were treated with a levonorgestrel-releasing IUD for either complex atypical hyperplasia or International Federation of Gynecology and Obstetrics (FIGO) grade I endometrioid endometrial cancer. Among the 21 patients with endometrial cancer, there was a 66.7% response rate. Thirty-seven patients had complex atypical hyperplasia, and they had a 90.6% response rate. The ability to offer a fertility-sparing option through targeting hormone receptors demonstrates the importance of biomarkers in guiding patient-centered treatments in gynecologic cancers [11].

In metastatic hormone receptor-positive endometrial cancer, hormonal suppression is a systemic treatment option. While response rates to hormonal therapy alone are low at less than 50%, it is a treatment option with an overall favorable side effect profile. Agents that can be utilized in this setting are high-dose progestins, aromatase inhibitors, and selective estrogen receptor modulators [12]. In the phase 2 PARAGON trial, 82 patients with hormonal therapy naive, hormone receptor-positive metastatic endometrial cancers were treated with anastrozole until progression of disease or unacceptable toxicity. The clinical benefit rate was 44% (95% CI: 34–55%) with a median duration of benefit of 5.6 months (95% CI: 3.0–13.7). Patients were considered ER- or PR-positive in this trial if their primary tumor or biopsy of recurrent disease demonstrated at least 10% of cells staining positive for ER and/or PR [4] (Table 1).

In the setting of uterine sarcomas, there are a group of low-grade sarcomas that can be treated in the advanced or recurrent setting with hormonal agents. The agents typically used are the same hormonal agents seen in endometrial cancer. In some cases, Gonadotropin-releasing hormone (GnRh) agonists, like Lupron, are added to more effectively target hormone receptors [13].

A subset of ovarian cancers is responsive to hormonal therapy including low-grade serous carcinoma, granulosa cell tumors, and endometrioid carcinoma. In these histologies, endocrine therapy is often utilized as a maintenance therapy or as treatment in the recurrent setting. Hormonal therapy is currently being investigated in the upfront setting but is not yet the standard of care. In low-grade serous carcinoma, response rates to standard cytotoxic therapy are low [14], so there is ongoing investigation into utilizing endocrine therapy as primary therapy in these patients given the good response rate and less toxic side effect profile. For example, the NRG study GY019 is a phase III randomized trial looking at endocrine therapy alone (letrozole 2.5 mg) following primary cytoreductive surgery for low-grade serous ovarian cancer versus carboplatin and paclitaxel followed by endocrine maintenance. The full results of this trial have not yet been published, but the study design reflects a move towards earlier use of endocrine therapy in this type of cancer [15].

#### 3.1.2. Laboratory Testing

Accurate assessment of hormone receptor expression is very important to determining eligibility for treatment with hormonal therapy. Although rigorous preanalytical variables (cold ischemic time and fixation time) are not required for gynecologic neoplasms, appropriate controls should be used, titrated and evaluated. In the uterus, the nonneoplastic glands, the endometrial stroma, and the endomyometrium are excellent positive controls. The myometrium has such excellent expression; it is recommended to be used as a positive control for hormone receptor testing in breast cancer. False negative results could be due to use of cautery during surgery, long cold ischemic time, non-optimal fixation or the utilization of different fixatives, such as ethanol, methanol, Bouin’s solution, Carnoy’s fluid, zinc formalin, and glyoxal.

The College of American Pathologists (CAP) recommends a modified version from the American Society of Clinical Oncology for the reporting of ER and PR for gynecologic cancers. The CAP guidelines for breast cancer set the cutoff for a positive test at 1% (unless otherwise specified for a particular trial) compared to a 10% cutoff in the PARAGON study [4]. The number of positive cells in percentage or in a discrete category (e.g., 10–20%) and the intensity of the staining (i.e., weak, moderate and strong) should be reported [16].

### 3.2. Human Epidermal Growth Factor Receptor-2 (HER2, ERBB2)

HER2 codes for a tyrosine kinase receptor that belongs to the epidermal growth factor receptor family. This protein plays a critical role in the signaling pathway that regulates cell division proliferation and differentiation. HER2 protein overexpression and/or gene amplification are the most used testing modalities to determine HER2 status. HER2 is overexpressed/amplified in endometrial serous carcinomas (30%), carcinosarcomas (16%), and clear-cell carcinomas (48%) [17,18,19,20]. It has been found that HER2 overexpression is associated in most cases with abnormal p53 expression. Therefore, HER2 testing is recommended in high-grade endometrial cancers (p53 abnormal) and HER2 testing is a reflex test in many institutions [17,18,19,20].

#### 3.2.1. Clinical Applications

The combination of trastuzumab with standard chemotherapy (carboplatin and paclitaxel) has shown an improvement in progression-free survival (PFS) and overall survival (OS) in HER2-positive endometrial carcinoma cases [21]. In a randomized phase II trial, 61 patients were assigned either standard of care carboplatin and paclitaxel or carboplatin, paclitaxel and trastuzumab. Median PFS was 8 versus 12.9 months (HR = 0.46; 90% CI, 0.28–0.76; *p* = 0.005) favoring the trastuzumab arm and median OS was 29.6 versus 24.4 months (HR = 0.58; 90% CI, 0.34–0.99; *p* = 0.046). However, scoring of HER2 in gynecologic tumors has not been standardized. In this randomized phase 2 clinical trial (NCT01367002) a 30% cutoff for HER2 protein overexpression by IHC was used to label the case as a positive case [22,23,24].

The DESTINY-PanTumor02 trial (NCT04482309) showed that trastuzumab-deruxtecan (T-DXd) has efficacy in treating patients with HER2-positive gynecologic tumors. In patients with tumors that are HER2-positive (3+), the response rates are as follows: endometrial 84.6%, cervical 75% and ovarian 63.6%. In Her2 equivocal (2+), the response rates are also excellent with response rates in endometrial (47.1%), cervical (40%) and ovarian (36.8%). The Food and Drug Administration (FDA) approved T-DXD for patients with unresectable or metastatic tumors that failed prior systemic therapy. The scoring of HER2 testing by IHC for Trastuzumab-Deruxtecan use is based on the enrollment criteria for the DESTINY-PanTumor02 trial (NCT04482309), which utilized the gastric cancer scoring system. Of note, heterogeneity in Her2 results can be seen between biopsy specimens and surgical specimens [5,10] (Table 1).

#### 3.2.2. Laboratory Testing

Unlike the pattern of complete, circumferential strong membranous staining in breast carcinoma, HER2 staining in endometrial cancers is usually lateral or basolateral with sparing of the apical portions of the tumor cells [19,22]. Due to the lack of a standardized scoring method in testing gynecologic malignancies, particularly the use of biopsy versus surgical specimens, the CAP suggests reporting HER2 using the enrollment criteria for the clinical trial NCT01367002, namely:

- Score 0 = no staining.
- Score 1+ (Negative): Incomplete, faint membrane staining in any proportion OR weak staining in <10% of tumor cells.
- Score 2+ (Equivocal): Strong complete or basolateral staining in <30% of cells OR weak-to-moderate staining in ≥10% of cells.
- Score 3+ (Positive): Intense complete or basolateral/lateral membrane staining in ≥30% of tumor cells [21,22,23,24].

**Figure 1:**
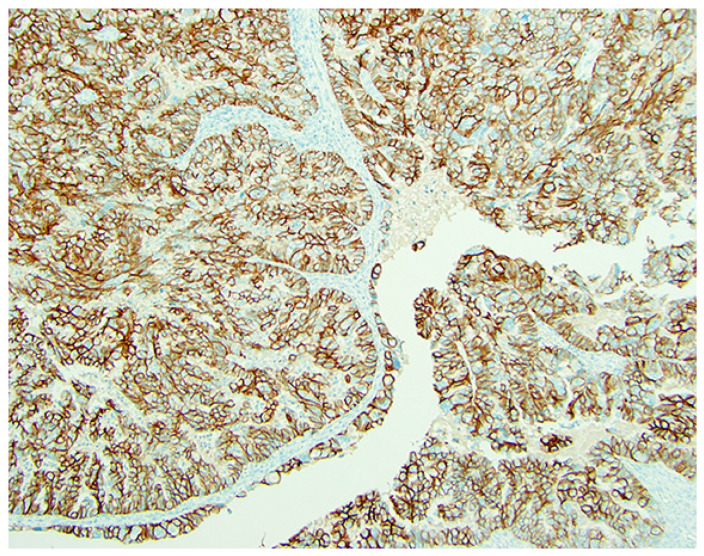
Her2 protein expression by immunohistochemistry. The gastric scoring system, namely the positive score (3+) as shown, is defined as tumor cluster (5 or more cells) with a strong, complete, basal lateral or lateral membrane staining in biopsies, while in surgical specimens, 10% is the cutoff for defining the positive score (×200). (Courtesy of Dr. M. Desouki).

There is no recommendation for HER2 testing regarding biopsy versus excisions in the DESTINY-PanTumor02 trial (NCT04482309). However, testing on multiple sites (biopsy, hysterectomy) is recommended [5,25]. In the gastric scoring system, a positive score (3+) is defined as a tumor cluster (5 or more cells) with a strong, complete, basal-lateral or lateral membrane staining irrespective of the percentage of the positive tumor cells in biopsies, while in surgical specimens, 10% is the cutoff. The same criteria (cluster versus 10%) defines the cutoffs for scores of 0, 1+ and 2+ HER2 scores [5,10]. (Table 1) (Figure 1).

For HER2 testing by in situ hybridization (ISH), all tumors with equivocal (2+) IHC scores should be reflexed to an ISH technique. For trastuzumab use, a HER2/CEP17 ratio of greater than or equal to 2 or a HER2 copy number of greater than or equal to 6 is considered positive for HER2 gene amplification. For ISH testing, at least 20 cells should be counted [22,24].

### 3.3. Mismatch Repair (MMR) Protein and Microsatellite Instability (MSI)

The treatment of endometrial cancer has been profoundly impacted by the identification of biomarkers allowing personalized models with incorporation of molecular, pathologic and clinical characteristics for risk stratification and treatment determinations. The Cancer Genome Atlas (TCGA) identified four categories of endometrial cancer: DNA Polymerase Epsilon (*POLE*) ultra mutated, MSI hypermutated, copy-number low, and copy-number high [26]. These categories demonstrate distinct clinical behavior that can be used to drive therapeutic decision making and prognostication. Testing for these categories and implementation in the clinical setting was further defined in the Proactive Molecular Risk Classifier for Endometrial Cancer (ProMisE) study which created a molecular classification system based on the TCGA groups [27]. These classifications are *POLE*-mutated, MMR-deficient (MMR-D), p53 wild-type (p53wt), and p53 abnormal (p53abn). These molecular classifiers were incorporated into the FIGO 2023 staging system for endometrial cancer, demonstrating their importance in the care of women diagnosed with endometrial cancer [28].

#### 3.3.1. Clinical Applications

MMR deficiency testing is extremely important for determining care for patients diagnosed with endometrial cancer. Loss of the expression of DNA MMR proteins (MLH1, MSH2, MSH6 and PMS2) can be associated with germline mutations in these genes or epigenetic changes resulting in the hypermethylation of the MLH1 promoter. While MLH1 loss is often due to methylation, loss of MSH2, MSH6, and PMS2 is more frequently associated with germline or somatic mutations. Patients who have tumors with loss of expression in these proteins without hypermethylation are recommended to undergo germline genetic testing for Lynch syndrome. Identification of patients with Lynch syndrome has a significant impact not only for treatment of their cancer but also implications for additional screening as well as cascade testing for family members. Approximately 3–5% of all endometrial cancers are associated with Lynch syndrome [29]. Approximately a third of endometrial carcinomas are MMR-D, while 14% of ovarian endometrioid carcinomas and 60% of ovarian clear-cell carcinomas are MMR-D. Lynch syndrome cases accounts for approximately 1% of ovarian carcinomas [30].

MMR testing has very significant clinical implications for primary therapy across all stages as well in the recurrent setting. The primary treatment for newly diagnosed uterine confined endometrial cancer is hysterectomy, bilateral salpingo-oophorectomy, and lymph node assessment. In young patients diagnosed with low-risk endometrial cancer (early stage, low grade), ovarian retention can be considered; however, in patients with a known diagnosis of Lynch syndrome, oophorectomy is generally recommended given a 6–12% risk of ovarian cancer [31].

In the setting of advanced stage primary disease or recurrent endometrial cancer, chemotherapy plus immunotherapy is the standard of care with patients with MMR-D. In two large, randomized trials comparing standard of care chemotherapy with carboplatin and paclitaxel to carboplatin and paclitaxel with an immune checkpoint inhibitor, a significant improvement in PFS was noted with the addition of immunotherapy with the most profound effect noted in the MMR-D cohort [6,7,32] (Table 1). Immunotherapy as a single agent can be utilized in the recurrent setting for treatment of MMR-D disease [33] or in combination with targeted agents like the tyrosine kinase inhibitor lenvatinib [34]. Across solid tumor types, MMR-D is associated with response to checkpoint blockade [35].

#### 3.3.2. Laboratory Testing

**Figure 2:**
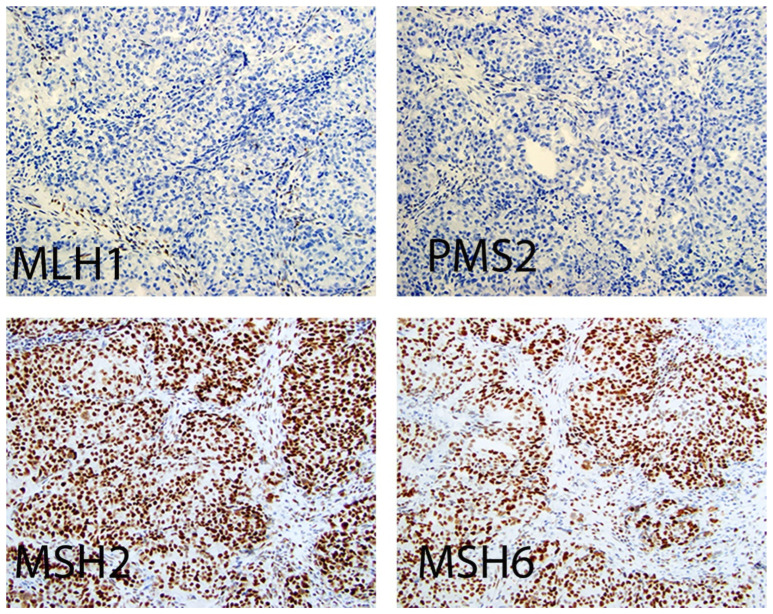
Mismatch repair protein expression by immunohistochemistry. Representative images showing loss of nuclear expression of MLH1 and PMS2 with intact expression of MSH2 and MSH6Note the internal positive control (stromal and inflammatory cells) is crucial in reporting (×200). (Courtesy of Dr. M. Desouki).

Universal MMR testing of all endometrial carcinomas by IHC and/or MSI is performed as part of initial diagnosis and treatment planning. MSI testing is performed using polymerase chain reaction (PCR) testing on formalin fixed, paraffin-embedded tumor tissue [36,37]. MMR proteins work in dimers where MLH1 loss accompanies PMS2 loss, while MSH2 loss is accompanied by loss of MSH6 in most cases (Figure 2). For reporting purposes, the MMR protein is reported as intact (normal) with positive nuclear staining or loss (abnormal) with negative nuclear staining. The internal positive control (stromal cells, smooth muscle cells, nonneoplastic epithelial cells or inflammatory cells) is crucial in reporting and should be stained positive for the tested to be valid [38]. Subclonal loss of one or more of the MMR proteins does occur when there are areas showing a complete loss of nuclear staining with intact staining in the remainder of the tumor. However, this should be distinguished from loss of the nuclear staining in the tumor cells with intact staining in the background hyperplasia or benign glands [39].

MLH1 loss may be due to epigenetic silencing such as MLH1 promoter methylation or mutations in the catalytic subunit of *POLE* in addition to somatic and germline Lynch syndrome. As is common in IHC testing, technical variables can impact interpretation. Missense mutations in the MMR genes may lead to intact protein expression, but a nonfunctional protein product. Aberrant expression can occur, which includes cytoplasmic, nucleolar or punctate nuclear staining, and these types of expression should be interpreted as abnormal and reflexed to the appropriate test [37]. It should also be kept in mind that intact expression of these proteins does not entirely exclude the possibility of Lynch syndrome, and this should be noted.

The pattern of MMR protein loss is a good surrogate for predicting which gene is mutated. For example, loss of MSH2 and MSH6 expression may suggest a germline mutation of the MSH2 gene. Variations in MLH1 and PMS2 should be sent for MLH1 promoter methylation studies. The presence of MLH1 promoter methylation suggests a sporadic tumor and its absence may be an indicative of a germline mutation. In the latter case, sequencing and/or large deletion/duplication testing of germline MLH1 is indicated [40].

Guidelines have been published by the CAP in collaboration with the Association for Molecular Pathology for the use of MMR and MSI testing as immunotherapeutic predictors. To determine the eligibility of endometrial cancer patients for immune checkpoint inhibitor therapy, MMR testing by either IHC or MSI by PCR/NGS are acceptable and considered to be guideline-recommended testing modalities [30]. The advantages of using IHC are relatively low cost, easy accessibility and relatively straightforward interpretation [37,41].

MSI is the presence of short repetitive sequences that are not present in the germline DNA due to defective DNA MMR. Testing is usually performed with at least five microsatellite markers, generally mononucleotide (BAT-25, BAT-26, NR-21, NR, 24, Mono-27) or dinucleotide repeat markers. The National Cancer Institute (NCI) panel includes (BAT-25, BAT26, D2S123, D5S346 and D17S250). Additionally, commercial kits for MSI testing that utilize five mononucleotide markers are available [42]. The MSI test is considered stable (MSS) if all the markers exhibit stability, low (MSI–L) if one of the mononucleotide markers exhibit instability, and as high (MSI-H) if two or more of the mononucleotide markers exhibit instability. In patients without MLH1 promoter methylation, MSI-H status raises suspicion for Lynch syndrome, which may be due to a germline mutation in one of the MMR genes or, rarely, an altered Epithelial Cellular Adhesion Molecule (EpCAM) gene [43]. MSI-L in gynecologic cancer is rarely a clinically actionable finding, and the focus is typically on MSS vs. MSI-H. As discussed above, MMR IHC is the primary testing; however, MSI is recommended for cases that show MSI-H to identify the probable germline or somatic mutation. The concordance between IHC and MSI testing by PCR/NGS is high (ranging widely from 58% to 93% in endometrial carcinomas) but not 100%, so these testing modalities are not completely interchangeable [44]. The same sample should be used if both IHC and MSI PCR testing are planned [42].

For the detection of MLH1 gene promoter methylation (epigenetic silencing), most laboratories utilize a methylation-specific real-time PCR to determine the presence of methylation. In contrast to colorectal cancer, B-Raf proto-oncogene, serine/threonine kinase (BRAF) mutations are extremely rare in endometrial cancer and testing is not recommended to be performed in gynecologic tumors [40,45].

### 3.4. P53 Protein and Tumor Protein p53 (TP53) Gene Mutations

P53 is a tumor suppressor protein that regulates cell proliferation, DNA repair, apoptosis and genetic stability. There can be inactivation of p53 or deactivation through binding proteins, such as E6 proteins from HPV high-risk genotypes, which result in dysregulation of cell growth and maturation.

#### 3.4.1. Clinical Applications

While p53 is not a direct therapeutic target like HER2 or PD-L1, its assessment by IHC is a powerful prognostic and diagnostic tool. The determination of p53 status is central to the molecular classification of endometrial and other gynecologic cancers. The TCGA identified a distinct molecular subtype of endometrial carcinomas with significant changes in PFS, copy-number high, and this category of endometrial cancer is associated with a poor prognosis [26]. *TP53* mutations are almost always present in high-grade serous tubo-ovarian carcinoma, endometrial serous carcinoma and carcinosarcoma as well as in a subset of endometrioid, clear-cell and undifferentiated carcinomas [37]. P53 can be utilized clinically to tailor endometrial cancer treatment as aberrant p53 expression is associated with greater benefit from the use of chemotherapy for adjuvant treatment [46,47].

The presence of a *TP53* mutation has been associated with improved OS and PFS in patients with advanced endometrial cancer treated with anti-angiogenic agents (bevacizumab) as compared to the mTOR inhibitor, temsirolimus, in combination with chemotherapy (PFS HR 0.48, 95% CI 0.31 to 0.75 and OS HR 0.61, 95% CI 0.38–0.98). There was no statistically significant difference in OS and PFS among patients with wild-type *TP53* [48].

#### 3.4.2. Laboratory Testing

P53 testing is a part of the diagnostic algorithm for endometrial carcinomas. IHC is a surrogate (but not perfect) marker for the detection of the *TP53* gene mutation with abnormal expression of the protein. P53 by IHC is a widespread reflex test for endometrial carcinomas to assist with determining subtype and molecular classification of the tumors. Additionally, p53 is a test performed in ovarian carcinomas, granulosa cell tumors with a high-grade transformation, mesenchymal tumors and vulvar intraepithelial lesions (differentiated VIN) and squamous cell carcinoma [37,49,50] to assist with diagnosis. It should be noted that abnormal p53 IHC does not equal a diagnosis of serous carcinoma. Subclonal abnormal p53 IHC pattern has been described in approximately 20% of endometrioid carcinomas, which may suggest secondary mutational events in MMR deficiency. Also, an abnormal p53 pattern may indicate mixed serous and endometrioid or clear-cell carcinoma [49,51]. Ovarian mucinous carcinoma may also show subclonal abnormal p53 patterns of intratumoral heterogeneity. In mucinous carcinoma, the abnormal overexpression of the p53 protein is in the basal layer of the neoplastic glands while sparing the superficial areas (terminal differentiation pattern) [52].

**Figure 3:**
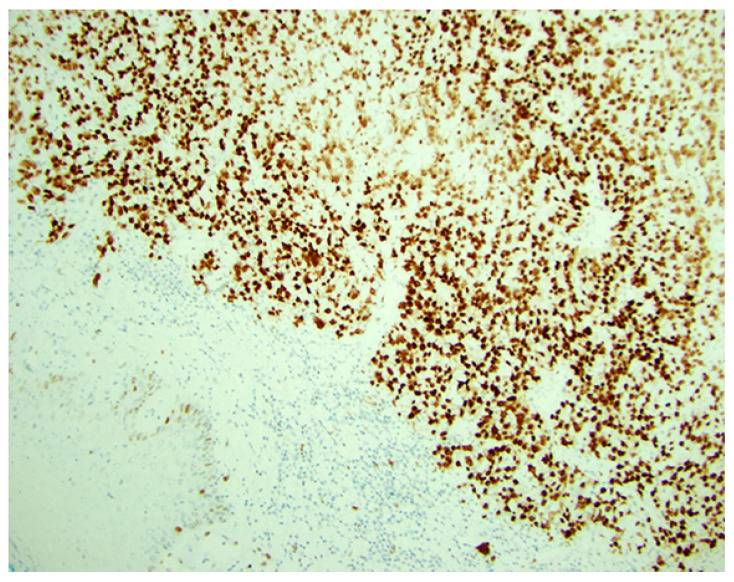
P53 expression by immunohistochemistry. Abnormal strong diffuse positive (mutant) type expression of P53 in ≥80% of the tumor cells (×200). (Courtesy of Dr. M. Desouki).

P53 abnormal (mutated) IHC patterns are defined as (1) overexpression (diffuse strong nuclear expression in ≥80% of the tumor cells) (Figure 3), (2) null type (complete absence of nuclear and cytoplasmic reactivity with unequivocal positive staining in internal controls, e.g., stroma, and lymphocytes) which arise due to a variety of mechanisms including insertions, deletions, nonsense or frameshift mutations of the *TP53* gene, and (3) aberrant cytoplasmic staining with or without nuclear staining often resulting from mutations at the nuclear localization domain that do not allow the p53 protein to enter the nucleus efficiently and therefore it is expressed in the cytoplasm [37,49,53]. In contrast, the normal (wild-type) pattern of reactivity denotes staining with variable intensity and cells do not usually have a mutated *TP53* gene.

The interpretation of p53 IHC can be impacted by multiple variables: (1) different clone used with the D07 clone showing the best performance and high interobserver agreement, (2) utilization of biopsy versus excision, (3) experience of the pathologists may impact the ability to recognize aberrant pattern of expression, (4) suboptimization of the antigen retrieval, (5) use of old archival material with poor fixation or other variables which may affect the interpretation. It is advised that cases with ambiguous p53 IHC expression are to be reflected to *TP53* gene sequencing [49,50,51].

In vulvar precursor and invasive carcinoma lesions, the abnormal (mutated) p53 IHC could be expressed as (1) basal overexpression with uniform strong diffuse nuclear expression in the basal layer, (2) parabasal diffuse overexpression, (3) absent/null with lack of nuclear or cytoplasmic expression, and (4) cytoplasmic expression with or without nuclear expression [54].

### 3.5. Programmed Death-Ligand 1 (PD-1) and PD Ligand 1 (PD-L1)

PD-1 and PD-L1 are expressed on T-lymphocytes, tumor cells and antigen-presenting cells. The expression of these immune checkpoint biomarkers is important in deciding whether to utilize immunotherapy [55]. PD-1, an immune checkpoint molecule, is a target for immunotherapies and treatment with immune checkpoint blockade has shown benefit in many tumor types [56].

#### 3.5.1. Clinical Applications

Inhibition of PD-1 (nivolumab and pembrolizumab) and PD-L1 (atezolizumab, durvalumab, and avelumab) has been proven effective in the treatment of many cancers including melanoma, non-small cell lung cancer (NSCLC), renal cell carcinoma. The use of immunotherapy has been incorporated into standard treatment for gynecologic cancers [55,57,58]. The FDA approved the immunotherapy Pembrolizumb for positive PD-L1 IHC stain (the 22C3 pharmDx assay [Dako, Carpinteria, CA, USA]). On the other hand, nivolumab, another immunotherapeutic drug, has been approved without this testing.

PD-L1 scoring is primarily incorporated into the treatment of locally advanced and recurrent/metastatic cervical cancer. For patients with recurrent/metastatic cervical cancer, the KEYNOTE 158 (NCT02628067) trial demonstrated an objective response rate (ORR) of 14.6% [59] with all responders demonstrating PD-L1 positivity defined as CPS ≥ 1. KEYNOTE 826 was a phase III randomized trial looking at adding immunotherapy to the standard regimen of carboplatin or cisplatin and paclitaxel with or without bevacizumab in patients with metastatic (83%) or persistent/recurrent (17%) cervical cancer. Patients were randomized to receive either pembrolizumab 200 mg/m^2^ or placebo with treatment. This study demonstrated increased PFS and OS with the addition of immunotherapy irrespective of the disease status (metastatic vs. local recurrence). Only a small subset of patients (11.2%) had a CPS < 1. While these patients appear to have less benefit from immunotherapy due to the limited number, it is not possible to determine efficacy definitively. Patients with PDL1 CPS of ≥10 were noted to have a higher response rate with pembrolizumab (21.4%) vs. (11.3%) in the placebo group [8] (Table 1).

Data from the KEYNOTE-A18 trial suggests that immunotherapy can also be combined with standard radiation and sensitizing chemotherapy in the setting of locally advanced, node-positive cervical cancer. Again, the majority (95%) of patients in this trial had PD-L1-positive (≥1 CPS) disease (94.3%) [60]. These data have shown the significant benefit immunotherapy can have for advanced and recurrent cervical cancer, with most pronounced benefit among women who are PD-L1-positive.

Immunotherapy as a single agent has shown limited response rate in the treatment of ovarian cancer [61] but has been effectively utilized in combination with other therapeutic agents [62]. Approximately 28–40% of ovarian cancers are PD-1- and PD-L1-positive [63]. Typically, PD-1 and PD-L1 testing has not been a routine part of ovarian cancer care but recently presented data from a randomized controlled trial in patients with recurrent, platinum-resistant epithelial ovarian cancer comparing the use of paclitaxel with or without bevacizumab plus placebo versus pembrolizumab demonstrated an improvement in PFS and OS with the use of pembrolizumab [64]. In total, 466 patients had tumors expressing PD-L1 with CPS ≥ 1 and the median PFS was 8.3 months (95% CI: 7.0, 9.4) in the pembrolizumab arm and 7.2 months (95% CI: 6.2, 8.1) in the placebo arm (HR 0.72 [95% CI: 0.58, 0.89]; *p*-value 0.0014). The median OS was 18.2 months (95% CI: 15.3, 21.0) in the pembrolizumab arm and 14.0 months (95% CI: 12.5, 16.1) in the placebo arm (HR 0.76 [95% CI: 0.61, 0.94]; *p*-value 0.0053). Based on these results the FDA has approved this regimen for use in the setting of PD-L1-positive ovarian cancer. It has also approved the PD-L1 IHC 22C3 pharmDx as a companion diagnostic in ovarian cancer.

#### 3.5.2. Laboratory Testing

**Figure 4:**
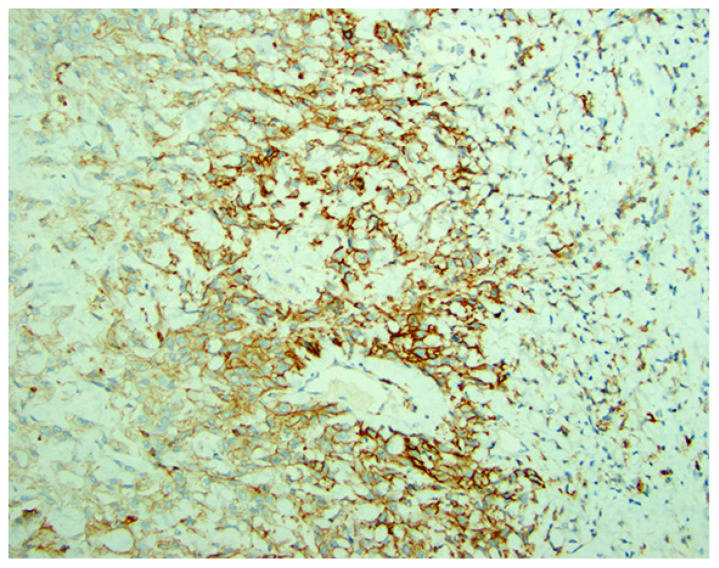
PD-L1 expression in cervical cancer case. Caption from an archival case where the Combined Positive Score (CPS) system is used. The ratio of PD-L1-positive cells (tumor cells, lymphocytes and macrophages) to the total number of PD-L1-positive and negative tumor cells is multiplied by one hundred (≥1 is considered positive) (×200). (Courtesy of Dr. M. Desouki).

While the expression of the PD-L1 protein has been approved to be a marker for response to immune checkpoint blockade, there is no consensus about the specifics of testing including method of testing, the clone used, or the cutoff for positive vs. negative. There are multiple IHC assays available for PD-L1 expression. However, several challenges exist with these assays, e.g., intratumoral heterogeneity, prohibitive cost, among others [65,66]. The most commonly used scoring systems for PD-L1 are (1) Tumor Proportion Score (TPS) where the percentage of membrane-positive (at any intensity) viable tumor cells are divided by the total number of PD-L1-positive and negative tumor cells multiplied by a hundred and (2) Combined Positive Score (CPS) where the ratio of PD-L1-positive cells (tumor cells, lymphocytes and macrophages) to the total number of PD-L1-positive and negative tumor cells is multiplied by a hundred (Figure 4). The TPS scoring system is applied mainly for lung cancer (advanced non-small cell lung cancer; NSCLC), while CPS is utilized for cervical, gastric, head and neck squamous cell carcinoma (SCC), and advanced triple negative breast cancer (TNBC). Different clones and cutoffs are used for different tumors which determine the eligibility for pembrolizumab. Table 2 summarizes PD-L1 scoring system and cutoffs used according to the tumor type and therapy utilized [67,68] (Table 2).

### 3.6. Folate Receptor Alpha (FOLR1)

FOLR1, a cell surface protein, plays a crucial role in transporting folate, which is essential for cell growth, into cells upon ligand binding. FOLR1’s role in cancer progression is multifaceted. Increased folate uptake due to FOLR1 overexpression may give tumors a growth advantage [69,70]. Additionally, FOLR1 might influence cell proliferation through other signaling pathways, requiring further investigation. This overexpression suggests FOLR1’s potential as a biomarker for targeted therapy.

#### 3.6.1. Clinical Applications

Within gynecologic cancer, FOLR1 has shown efficacy in the treatment of platinum-resistant ovarian cancer, where response rates are typically less than 20%. FOLR1 has been utilized as a target in a novel category of drug called antibody drug conjugates (ADCs), which have shown promise across tumor types [69,70,71]. Mirvetuximab soravtansine is an antibody drug conjugate targeting FOLR1 with FDA approval for the treatment of platinum-resistant, FOLR1-expressing ovarian cancer with a response rate of 42% in the phase III randomized MIRASOL trial [9] (Table 1). A prior phase II study utilizing the same staining threshold, the SORAYA study, had shown an ORR of 32.4% (95% CI, 23.6 to 42.2), suggesting significant benefit within the FOLR1 high population [72]. The FORWARD I trial was a randomized phase III study reported that there is no statistically significant difference in outcomes between mirvetuximab soravtansine and standard of care chemotherapy. In this trial, even within the FOLR1 high subset of patients. The use of propensity scoring to determine FOLR1 positivity as opposed to membranous FOLR1 staining may have allowed for the inclusion of patients with lower FOLR1 expression and impacted the findings [69]. This highlights the significant clinical implications of testing methodology for FOLR1. FOLR1 targeting is also being actively investigated in endometrial cancer through the use of alternative antibody drug conjugates.

#### 3.6.2. Laboratory Testing

**Figure 5:**
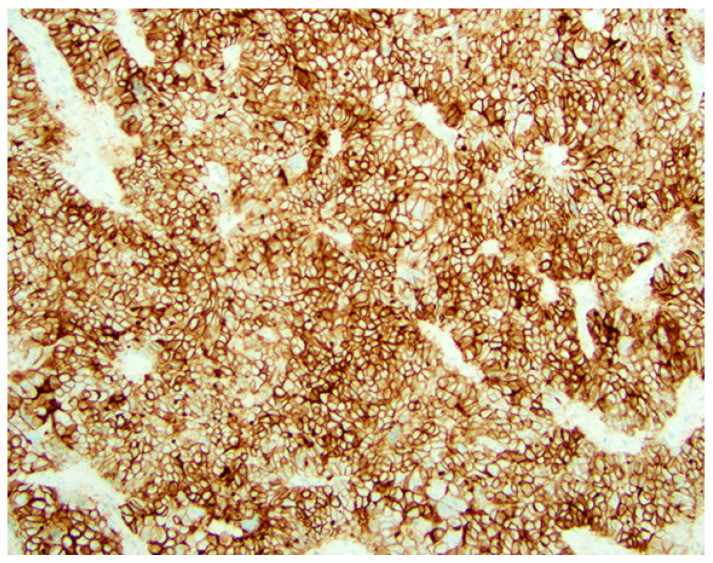
FOLR1 immunohistochemistry in a high-grade serous ovarian carcinoma. A positive result, as per the PS2+ scoring method, requires ≥75% of viable tumor cells showing moderate (2+) or strong (3+) membranous staining (×200). (Courtesy of Dr. M. Desouki).

The Ventana FOLR 1-2.1 RxDX Assay (Roche, Boston, MA, USA) and IHC-based test utilizing FOLR1-2.1 clone have been FDA approved as the companion diagnostic for this treatment indication. A positive result is defined as 75% or more tumor cell staining with moderate or strong intensity with normal fallopian tube as a control [73] (Figure 5). The patients included in phase III randomized MIRASOL trial had high FOLR1 tumor expression defined as ≥75% of cells with ≥2+ membranous staining intensity [9]. The FORWARD I trial utilized a broader inclusion criteria for FOLR1 positivity. In this trial it was defined as ≥50% of tumor cells with any FOLR1 membrane staining visible at ≤×10 microscope objective, 50–74% and ≥75% representing medium and high expression, respectively [69].

### 3.7. DNA Polymerase Epsilon (POLE) Mutation

*POLE* encodes a major catalytic and proofreading subunit of the DNA polymerase enzyme complex. The function of the exonuclease is to locate and replace errors in the new DNA strands that failed to pair with the parent strand [74].

#### 3.7.1. Clinical Applications

Mutations in *POLE*, specifically the exonuclease domain, such as P286R and V411L, will cause ultra mutated tumors. In gynecology this is primarily seen in endometrioid carcinoma. Endometrial tumors with *POLE* mutations are classified as “*POLE* ultra mutated”, which are molecularly defined groups of tumors with thousands of somatic mutations constituting 9% of endometrial cancer cases. *POLE*-mutated tumors usually have a favorable prognosis and often treatment can be de-escalated in this cohort [26].

#### 3.7.2. Laboratory Testing

The clinicopathologic features of endometrial cancers harboring POLE mutations include endometrioid phenotype (84%), less than 50% myometrial invasion (56%), lymphovascular invasion (25%) and lymph node metastasis (29%) [75]. POLE mutant tumors are often infiltrated by cytotoxic and helper T cells and can have PD-1 expression which makes them responsive to immune checkpoint inhibitors. However, given their excellent prognosis and overall minimal response rates, systemic treatment is rarely needed. The OS and PFS in patients with POLE mutations are very often compared to other molecular types of endometrial cancer (HR, 0.57; 95% CI, 0.40–0.79), s (HR, 0.37; 95% CI, 0.26–0.53), respectively [76].

*POLE* testing is performed on formalin-fixed, paraffin-embedded tissue using next-generation sequencing (NGS) to detect hotspot somatic rather than germline alterations. One of the methods to detect the exonuclease domain of *POLE* is by using PCR amplification and Sanger sequencing [77]. Conventional Sanger sequencing screens exons 9–14 of the gene containing most of the hot spot mutations. PCR on the tumor DNA uses primers flanking these exons followed by sequencing and analysis for the known pathogenic POLE hotspot mutations (p.P286R, p.S297F, p.V411L, p.A456P, and p.S459F). The case is considered positive for the POLE mutation if a heterozygous variant equivalent to the pathogenic hotspot is identified [78]. Droplet digital polymerase chain reaction (ddPCR) has been introduced as an accurate, rapid, cost-effective method to detect POLE mutations [79,80].

### 3.8. Circulating Biomarkers

Detection of circulating biomarkers is increasingly being used to guide therapeutic decision making and surveillance in the treatment of gynecologic cancer. Circulating tumor DNA (ctDNA) or liquid biopsy is a promising modality that is being incorporated into clinical practice, although its optimal usage is still being determined. ctDNA are fragments of short (~146 bp) single- or double-stranded DNA that are released in the bloodstream by the tumor cells. The half-life is approximately 15 to 150 min [81].

Liquid biopsy in biofluids include blood, cerebrospinal fluid, saliva, urine, ascitic fluid, etc., ctDNA is isolated from the body fluid to detect its presence or absence in a variety of gynecologic tumors including ovarian, cervical and endometrial cancers [82]. The technique has multiple advantages compared to solid tumor biopsy which includes cost effectiveness, minimally invasive, highly sensitive and relatively rapid method [83]. However, limitations of this method exist which include low volume of ctDNA which makes it difficult to be detected, limited data regarding shedding of ctDNA across tumor types, and lack of widely accepted standardized protocols [84]. In clinical practice, ctDNA can be used for diagnosis of cancer at an early stage, detection of recurrence, assessment of therapy response and for treatment selection.

Currently this is used in the setting of locally advanced, metastatic and recurrent tumors. Multiple trials have used ctDNA to identify and monitor response to therapy. It has been reported that persistence of ctDNA after treatment is associated with worse PFS. Given this, it may be able to help identify patients with high risk of recurrence who may benefit from additional therapy [85,86,87].

### 3.9. Challenges and Limitations in Biomarker Testing

In addition to utilizing biomarkers in diagnosis, their testing in the pathology laboratory has become a key component of management of gynecologic cancers. In addition to guiding treatment decisions, markers are used to determine prognosis and likelihood of response to treatment [88]. One of the basic testing modalities utilized is IHC which requires multistage procedures including, but not limited to, tissue processing and antigen retrieval [89]. The advantages of using IHC include relatively low-cost, easy scoring, accessibility and utilization of light microscopy [90]. However, challenges and limitations do exist, which include reaction bias when performing certain steps such as antigen retrieval, specimen fixation, utilization of different clones, availability of controls, cross reaction between antigens (false positive), and interpretation bias among others. The skills of the personnel performing and interpreting IHC stains are crucial in titrating the antibodies, interpreting the results in the correct lesional cells, and identifying specific subcellular localization [88,89,90].

ISH technology utilizes DNA or RNA probes that anneal with the target sequence. The probes are labeled with markers to allow detection of hybridization through different techniques including fluorescence. The ISH technique is commonly used to diagnose genetic diseases as well as understand the pathogenesis of many malignancies [91]. ISH technology is not immune from challenges and limitations and requires skilled personnel to perform and interpret. The utilization of ISH is primarily limited by the availability of the probes. Additionally, the size of genomic aberrations can be a limiting factor. One of the limitations and the challenges for utilizing both IHC and ISH is the consistency in reporting and utilization of the results in patient management. Therefore, several modifications have been introduced over time; for example, the combined efforts of the American Society of Clinical Oncology (ASCO) and CAP in refining and modifying the guidelines for HER2 testing in breast cancer [88].

## 4. Conclusions

Biomarker testing is a key component of diagnosis, and treatment planning in gynecologic cancer and markers routinely tested by pathologists have a profound impact on patient care. Utilizing appropriate methodology to assess biomarker expression is crucial as the testing type needs to match the available data to accurately determine whether a patient is eligible for a targeted therapy or to prognosticate appropriately. Biomarker testing will continue to play an important role in the treatment of gynecologic malignancies and will continue to expand with the ongoing trend towards personalized medicine in these cancers. It is critically important for practicing pathologists to be familiar with preanalytic variables (cold ischemic time if any, fixation used and fixation time) and assay selection for different markers (IHC, PCR, molecular testing) to determine the most robust, clinically applicable and cost-effective testing modality. Appropriate test interpretation, scoring systems and cutoffs used should be utilized to maximize clinical utility of biomarker testing.

## Figures and Tables

**Table 1:** Summary of representative clinical trials utilized the biomarkers discussed in the manuscript *.

Source	Eligibility	Biomarker	Primary Endpoint	Result	Testing Methodology/Companion Diagnostic
PARAGON Trial [4]	Recurrent/metastatic endometrial cancer that is ER- and/or PR-positive	ER/PR	Clinical benefit	44%	IHC ER/PR Positivity defined as at least 10% nuclear positivity
DESTINY-PanTumor02 [5]	Locally advanced, metastatic or unresectable solid tumors (excluding breast, colorectal, gastric and non-small cell lung) with HER2 2+ or 3+	HER2	Objective response rate	Endometrial: 57.5% Ovarian: 45% Cervical: 50%	IHC Her2 utilizes gastric cancer scoring. Both biopsy and resection specimens were utilized **
GY018 [6]	Advanced or recurrent endometrial cancer, regardless of MMR status	MMR	PFS	dMMR: 74% vs. 38% (HR 0.3, 95% CI 0.19 to 0.48) pMMR: 13.1 vs. 8.7mo (HR 0.54, 95% CI 0.41–0.71)	IHC for MMR proteins
RUBY [7]	Advanced or recurrent endometrial cancer, regardless of MMR status	MMR/MSI	PFS, OS	dMMR: PFS 61.4% vs. 15.7% (HR = 0.28, 95% CI, 0.16 to 0.50); OS 22.6% vs. 53.8% (HR = 0.32, 95% CI 0.17–0.63) pMMR: PFS 28.4% vs. 18.8% (0.76; 95% CI, 0.59 to 0.98); OS 50.5% vs. 59.2% (HR = 0.79, 95% CI 0.60–1.04, nominal *p* = 0.0493)	Primarily IHC, but next-generation sequencing and PCR permitted
KEYNOTE 826 [8]	Persistent, recurrent or metastatic cervical cancer	PD-L1	PFS, OS	PDL-1 CPS ≥ 1: PFS 10.4 m vs. 8.2 m 0.62 (95% confidence interval [CI], 0.50 to 0.77; *p* < 0.001); intention to treat population: 10.4 months vs. 8.2 months (hazard ratio, 0.65; 95% CI, 0.53 to 0.79; *p* < 0.001); OS 50.4% vs. 40.4% (hazard ratio, 0.67; 95% CI, 0.54 to 0.84; *p* < 0.001)	PD-L1 IHC to determine CPS, predetermined stratification to CPS <1 vs. ≥1
MIRASOL [9]	Platinum-resistant, high-grade serous ovarian cancer	FOLR1	PFS	5.62 months (95% confidence interval [CI], 4.34 to 5.95) vs. 3.98 months (95% CI, 2.86 to 4.47) (*p* < 0.001)	VENTANA FOLR1 RxDx Assay utilizing the PS2+ scoring method (≥75% viable tumor cells with 2+ or 3+ staining intensity)

* P53 is not included in the table because it is a prognostic marker rather than a therapeutic target. ** Positive Her2 (3+) is defined as tumor cluster of 5 or more cells exhibiting a strong, complete, basal lateral or lateral membrane staining in biopsies, while in surgical specimens, 10% is the cutoff for defining the positive score [5,10].

**Table 2:** PD-L1 scoring system and cutoffs used according to the tumor type and therapy used [67,68].

Tumor Type	Therapy Used	PD-L1 Scoring System	PD-L1 Cutoff
Cervical Cancer	Pembrolizumab (KEYTRUDA)	CPS	≥1
GEJ adenocarcinoma	Pembrolizumab	CPS	≥1
HNSCC	Pembrolizumab	CPS	≥1
Ovarian cancer	Pembrolizumab	CPS	≥1
NSCLC	Pembrolizumab	TPS	≥1
ESCC	Pembrolizumab	CPS	≥10
TNBC	Pembrolizumab	CPS	≥10
NSCLC	Cemiplimab	TPS	≥50

PD-L1 Programmed death-ligand 1; NSCLC non-small cell lung cancer; TPS tumor proportion score; GEJ gastroesophageal junction; TNBC triple-negative breast cancer; ESCC esophageal squamous cell carcinoma; HNSCC head and neck squamous cell carcinoma; CPS combined positive score.

## Data Availability

No new data were created or analyzed in this study.

## Abbreviations

The following abbreviations are used in this manuscript:

ADCs	Antibody-drug conjugates
ASCO	American Society of Clinical Oncology
BRAF	B-Raf proto-oncogene, serine/threonine kinase
CAP	College of American pathologists
CPS	Combined Positive Score
ctDNA	Circulating tumor DNA
ELISA	Enzyme-Linked Immunosorbent Assa
EpCAM	Epithelial Cellular Adhesion Molecule
ER	Estrogen receptors
ESCC	Esophageal squamous cell carcinoma
FIGO	International Federation of Gynecology and Obstetrics
FDA	Food and Drug Administration
FOLR1	Folate receptor alpha
GEJ	Gastroesophageal junction
GnRh	Gonadotropin-releasing hormone
GOG	Gynecologic Oncology Group
HER2	Human Epidermal growth factor Receptor-2
HNSCC	Head and neck squamous cell carcinoma
IHC	Immunohistochemistry
ISH	in-situ hybridization
IUD	Intrauterine device
MMR	Mismatch Repair
MSI	Microsatellite Instability
NCI	National Cancer Institute
NCCN	National Comprehensive Cancer Network
NSCLC	Non-small cell lung cancer
ORR	Objective response rate
OS	Overall survival
PCR	Polymerase Chain Reaction
PD-L1	Programmed death-ligand 1
PEComa	Perivascular epithelioid cell tumors
PFS	progression free survival
*POLE*	polymerase epsilon
PR	Progesterone receptors
ProMisE	Proactive Molecular Risk Classifier for Endometrial Cancer
TCGA	The Cancer Genome Atlas
TNBC	Triple negative breast cancer
*TP53*	Tumor Protein p53
TPS	Tumor Proportion Score
VIN	Vulvar intraepithelial lesions
